# Sonographic localisation of lymph nodes suspicious of metastatic breast cancer to surgical axillary levels

**DOI:** 10.1002/jmrs.840

**Published:** 2024-11-17

**Authors:** Michelle Fenech, Tracey Burke, Grace Arnett, Alisha Tanner, Natasha Werder

**Affiliations:** ^1^ College of Clinical Sciences, Health, Medical and Applied Sciences CQUniversity Brisbane Queensland Australia; ^2^ Department of Medical Imaging Royal Brisbane and Women's Hospital Brisbane Queensland Australia

**Keywords:** Axilla levels, axilla ultrasound, axillary lymph node groups, breast cancer, lymph node sonography, nodal metastasis

## Abstract

The axillary lymph node (LN) burden of breast cancer patients guides multidisciplinary management and treatment regimes. Sonographic imaging is used to identify the presence, number and location of axillary LNs suspicious of malignancy and used to guide nodal fine needle aspirations and biopsies. Axillary LNs suspicious of harbouring breast cancer metastasis can be localised to three surgical axillary levels, numbered according to their location relative to the pectoralis minor muscle and lymph flow. To sonographically identify and localise suspicious axillary LNs, an understanding of the axillary anatomy, muscular sonographic landmarks, surgical axillary levels, and the sonographic technique to image and distinguish between benign and suspicious LNs is required.

## Graphical abstract

Sonographic imaging is used to identify the presence, number and location of malignant lymph nodes. Axillary lymph nodes suspicious of harbouring breast cancer metastasis can be localised to three surgical axillary levels. This paper will unpack the axillary anatomy, muscular sonographic landmarks, surgical axillary lymph node levels and the sonographic technique to image and distinguish between benign and suspicious lymph nodes.

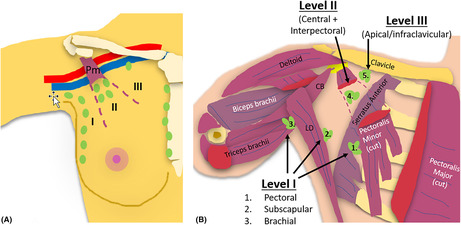

## Introduction

Lymph node (LN) metastasis is the most important predictor of overall recurrence and survival of breast cancer.^1^ Accurate assessment of axillary LN involvement is an essential component in staging breast cancer and deciding on the appropriate treatment: systemic therapy, extent of surgery, reconstruction options and radiation therapy requirements.^2^ Although the American Joint Committee on Cancer (AJCC) does not require imaging studies to assign clinical LN categorisation for breast cancer staging, sonographic assessment of the axilla is commonly utilised in preoperative and surveillance stages. Ultrasound imaging is used to identify the presence, number and location of LNs suspicious of malignancy and guide fine needle aspiration cytology or core needle biopsies.^3^, ^4^, ^5^, ^6^

Sonographic assessment of axillary LNs in breast cancer patients requires knowledge of the axillary anatomy, LNs, axillary surgical levels and the sonographic technique to image, localise and discriminate between benign and suspicious axillary LNs. There is currently a lack of published systematic sonographic protocol to image axillary LNs. Subsequently, sonographic axillary LN assessment can be variably performed, and documentation of suspicious axillary LN appearances and locations can be inconsistent. Sonographic localisation of axillary LNs should allow correlation to landmarks used in computed tomography (CT), positron emission tomography CT (PET CT) and magnetic resonance (MR) imaging. This paper will unpack the anatomy of axillary LNs of the breast, axillary surgical levels, the sonographic technique to image and discriminate between benign and suspicious axillary LNs and localise them to appropriately inform patient management, including any required surgical planning.

## Anatomy of the Axilla

**Figure 1:**
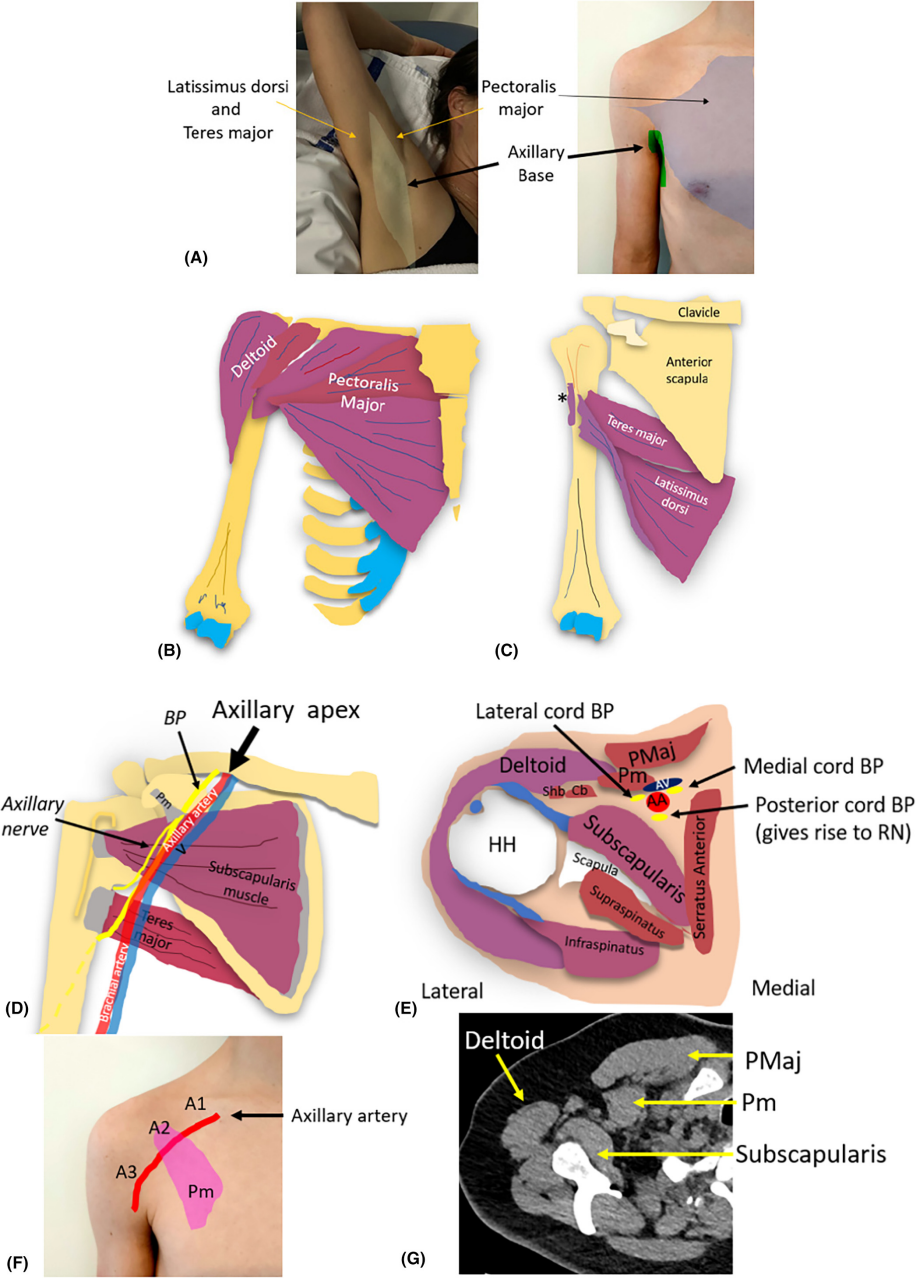
The axilla. (A) Photos of the axillary base with the arm above the head and by the side. When the arm is above the head, the anterior and posterior axillary folds can be identified. The anterior axillary fold is formed by the pectoralis major muscle which inserts onto the lateral lip of the intertubercular groove of the humerus. The posterior axillary fold is formed by the latissimus dorsi and teres major muscles and tendons. The muscles are posteriorly located but insert onto the floor and the medial lip of the intertubercular groove of the humerus. (B and C) Diagrams of the locations of the pectoralis major, teres major and latissimus dorsi that comprise the anterior and posterior axillary folds, and their distal insertions. (D) Axillary apex, (E) Transverse cross section of the axilla with the arm placed by the side. (F) The three parts of the axillary artery (A1, A2 and A3) relative to the pectoralis minor muscle. (G) Axial computed tomography (CT) image with arm above head. A1, Axillary artery part 1; A2, Axillary artery part 2; A3, Axillary artery part 3; AA, axillary artery; AV, axillary vein; BP, brachial plexus; Cb, coracobrachialis muscle; Pm, pectoralis minor muscle; PMaj, pectoralis major muscle; RN, radial nerve; Shb, short head of biceps brachii muscle.* Distal insertion point of the pectoralis major muscle onto the lateral lip of the intertubercular groove.

To sonographically image the axilla, its anatomy must be understood. The axilla is a complex pyramidal shaped region between the lateral upper thoracic wall and the medial aspect of the arm.^7^ Neurovascular structures and lymphatic channels pass through the fat filled axilla, between the neck, thorax and upper limb.^8^ The axilla has a base, apex and four walls; anterior, medial, lateral and posterior.^8^ Anatomically, the concave shaped axillary base (armpit), formed by skin and axillary fascia, comprises the axillary caudal end when the arm is by the side.^9^ The base is bounded anteriorly by the anterior axillary fold, formed by the lower border of the pectoralis major muscle. Posteriorly, the base is formed by the posterior axillary fold, formed by the latissimus dorsi and teres major muscles and tendons^9^ (Fig. 1).

The axillary apex (axillary inlet, thoracic outlet or cervico‐axillary canal) is the most proximal part of the axilla, closest to the neck.^10^ Bounded by the clavicle, the superior border of the scapula and the lateral border of the first rib, it contains fat, and LNs.^11^, ^12^ The inlet (or base) allows passage of the axillary artery and vein, brachial plexus and lymph vessels into or out of the axilla.^12^

Four axillary walls, formed by musculoskeletal structures, can all be identified sonographically. The anterior axillary wall has two layers; superficial and deep. The pectoralis major muscle forms the superficial layer.^12^ The deeper layer of the anterior axillary wall is formed by the pectoralis minor and subclavius muscles, clavipectoral fascia and the suspensory ligament of the axilla (inferior to the pectoralis minor muscle).^9^ The clavipectoral fascia connects the subclavius and pectoralis minor muscles.^9^ The chest wall comprised of the serratus anterior muscle, ribs 1–5, and their associated intercostal muscles, forms the medial axillary wall.^8^ The lateral axillary wall is formed by the intertubercular groove of the humerus, proximal tendons of the long and short heads of the biceps brachii and the coracobrachialis muscle.^11^ The subscapularis, teres major and latissimus dorsi muscles from superior to inferior form the posterior axillary wall.

### Axillary contents

**Figure 2:**
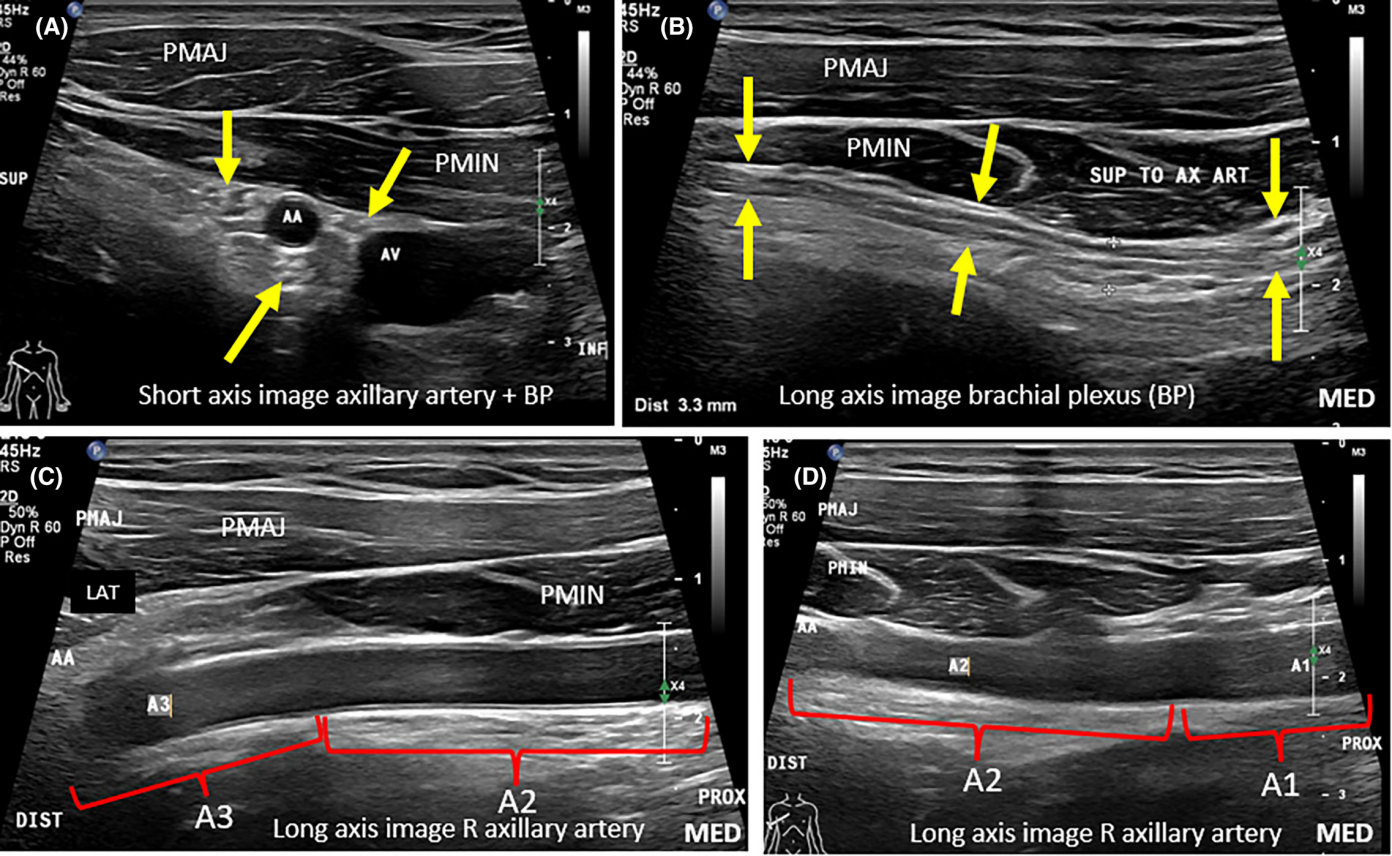
Structures that pass through the axilla. (A). Sonographic image demonstrating the short axis of the axillary artery and brachial plexus (BP) cords (yellow arrows). Transducer position outlined by body marker. (B). Image demonstrating the long axis of one of the brachial plexus cords (yellow arrow). (C). Sonographic image demonstrating the long axis of parts 3 (A3) and 2 (A2) of the right (R) axillary artery. Part 3 is located lateral to the pectoralis minor muscle, and part 2 is located deep to the pectoralis minor muscle. (D). Sonographic imaging demonstrating part 2 (A2) and part 1 (A1) of the axillary artery. AA, axillary artery; AV, axillary vein; MED, medial aspect of long‐axis images; PMAJ, pectoralis major muscle; PMIN, pectoralis minor muscle.

Major structures within the axilla include the axillary artery and vein, the brachial plexus (BP), lymphatic channels and LNs. The axillary vessels are good sonographic landmarks. The axillary artery, the continuation of the subclavian artery, becomes the axillary artery once it extends beyond the lateral edge of the first rib.^11^ It has three parts relative to the pectoralis minor muscle, numbered from proximal to distal, indicating the direction of arterial flow: part 1, proximal to pectoralis minor, part 2, deep to pectoralis minor, and part 3, lateral to pectoralis minor^13^ (Fig. 2).

Likewise, the axillary vein continues proximally from the brachial vein, and duplication or triplication of the axillary vein can be encountered.^11^ Tributaries of the axillary vein correspond to branches of the axillary artery but also include the cephalic vein. The axillary artery gives rise to branches; the thoracodorsal, lateral thoracic, subscapular, and anterior and posterior circumflex humeral arteries.^12^ The axillary artery becomes the brachial artery once it extends beyond the inferior edge of the teres major muscle.^11^ The brachial plexus (BP) consists of five components from proximal to distal: roots, trunks, divisions, cords and five main branches. The BP cords and branches in the axilla should be appreciated sonographically and must not be confused for pathology. Minor structures within the axilla include the intercostobrachial, long thoracic and thoracodorsal nerves, the thoracodorsal artery and the long thoracic vein, many of which can be underappreciated on imaging.^14^

## Lymphatic Drainage and Regional Lymph Nodes of the Breast

The breast lymphatics are separate from that of the underlying torso.^7^ There are four classically described intercommunicating lymphatic plexuses in the breast: two superficial and two deep.^15^ The superficial plexuses are located in the dermal and subcutaneous regions and include the areolar and subareolar (Sappey's) plexus.^15^ The deep lymphatic plexuses lie within the pectoralis major muscle fascia (fascial plexus) and the lobes and ducts of the mammary gland (glandular plexus).^15^ The lymphatic channels accompanying lactiferous ducts in the glandular plexus, communicate with the subareolar plexus via perforating lymphatic channels.

**Figure 3:**
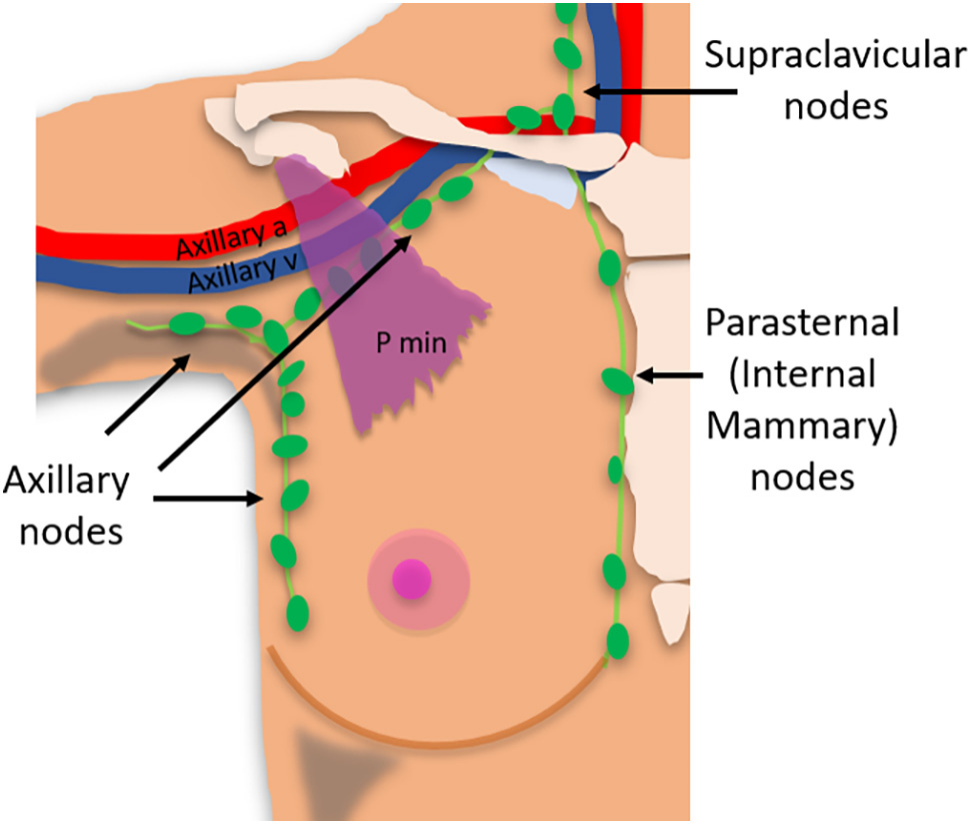
Regional lymph nodes of the breast which include: Intramammary, axillary, parasternal (internal mammary) and supraclavicular lymph nodes.

Regional LNs of the breast, those most likely to be involved in metastatic breast cancer, include intramammary, axillary, internal mammary (parasternal) and supraclavicular nodal chains.^16^ The superficial lymphatic plexuses of the breast drain directly to the axillary LNs.^7^ The deep lymphatic breast plexuses drain mostly into the axillary LNs, but may initially drain into intramammary LNs. Additionally, a variable degree of deeper breast tissue of the medial breast drains into the parasternal (internal mammary) LNs^15^ (Fig. 3).

Intramammary LNs, which reside within the breast tissue, are most commonly found in the upper outer quadrant and are considered axillary LNs for AJCC cancer staging.^16^ The internal mammary (also called parasternal) nodal chain extends from the first through to the sixth intercostal space, along the edge of the sternum.^16^ These nodes travel along the internal mammary (thoracic) artery and vein, deep to the parietal pleura.^15^ Supraclavicular LNs, although considered neck LNs, are a site of regional recurrence of breast cancer.^15^ They are located in a triangle delineated by the omohyoid muscle and tendon supero‐laterally, the internal jugular vein (IJV) medially, and the clavicle and subclavian vein inferiorly.^16^

## Surgical Levels of the Axillary Lymph Nodes

**Figure 4:**
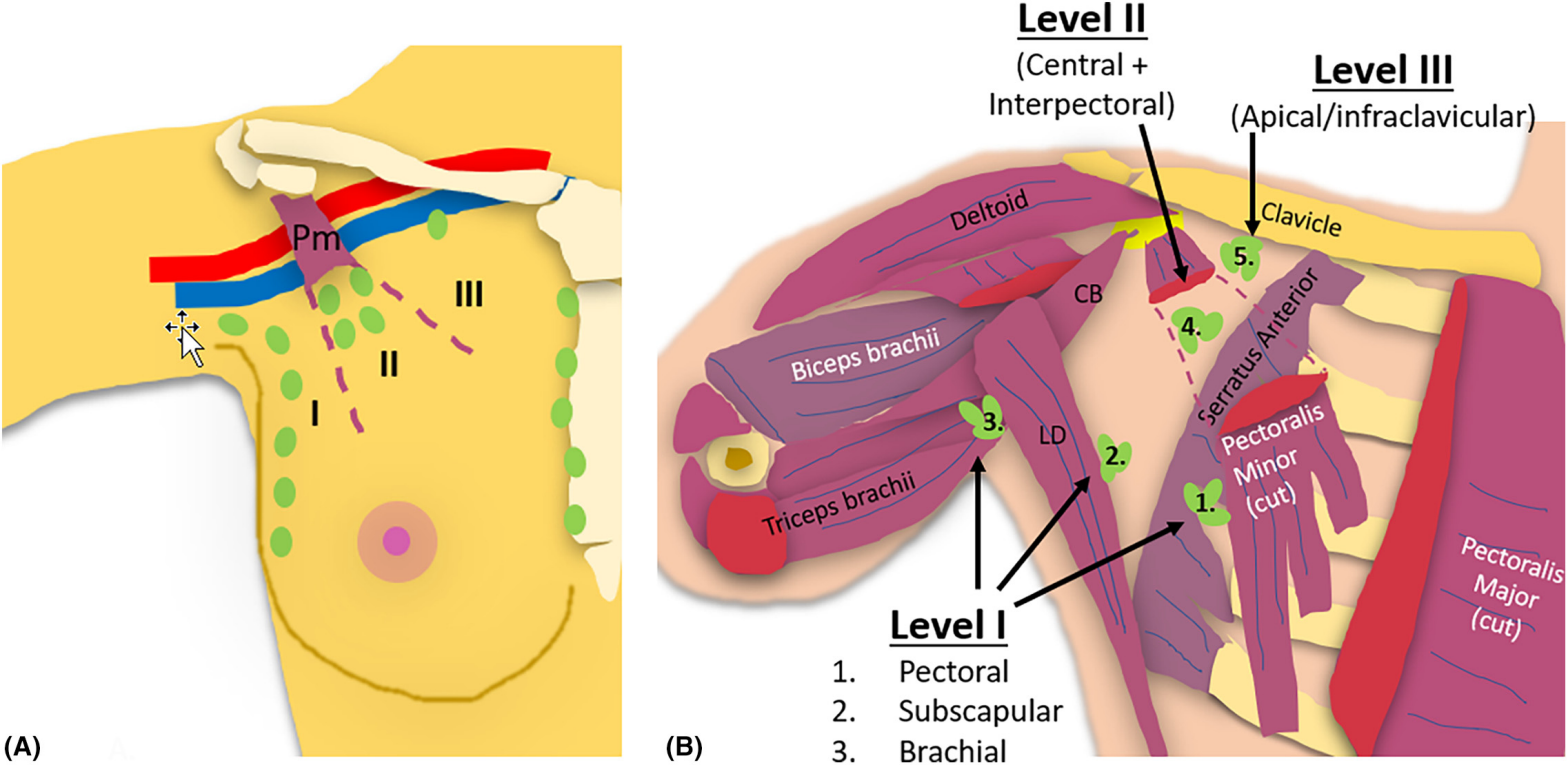
(A) Three surgical levels of axillary lymph nodes, relative to the pectoralis minor muscle (Pm). (B) Five groups of axillary lymph nodes. Level I contain 3 main groups of axillary nodes: 1. Anterior (pectoral) 2. Posterior (subscapular) 3. Lateral (brachial), Level II contains the 4. Central (and interpectoral nodes) and Level III contains the Apical (Infraclavicular) nodes (5).

As breast lymphatic drainage predominantly drains to axillary LNs, they are the main destination for breast cancer LN metastasis.^15^ Embedded within fibrofatty tissue, axillary LNs are subdivided into five anatomical sub‐groups based on their location: anterior (pectoral or external mammary), posterior (subscapular), lateral (humeral or brachial), central and apical (infraclavicular).^17^ These axillary LN sub‐groups are located within three different surgical axillary levels which are used to define LN locations. The three surgical levels (Level I, II and III) were defined by Berg (1954), hence, also called Berg's levels are located relative to the pectoralis minor muscle.^18^, ^19^ They are numbered according to the direction of lymph flow (towards midline). It must be noted that the axillary levels are numbered inversely relative to axillary artery levels (Fig. 4).

Level I of the axilla includes LNs infero‐lateral to the pectoralis minor muscle (Berg's Level 1). Level II of the axilla includes LNs between the medial and lateral borders of the pectoralis minor muscle (Berg's Level 2), and Level III defines LNs supero‐medial to the pectoralis minor muscle (Berg's Level 3).^7^ Lymph drainage typically proceeds from level I to level II to level III and then into the thorax.^20^ Any axillary LN level may receive lymphatic drainage from the breast, and hence the sentinel LN (initial LN to which a primary tumour drains) may potentially be located in any level, although most sentinel LNs are located in level I.^21^, ^22^ Imaging evidence of LNs suspicious of malignancy at level I and II warrants further imaging and assessment of level III, internal mammary and supraclavicular LNs for involvement.^23^

### Surgical axillary Level I (low axilla)

**Figure 5:**
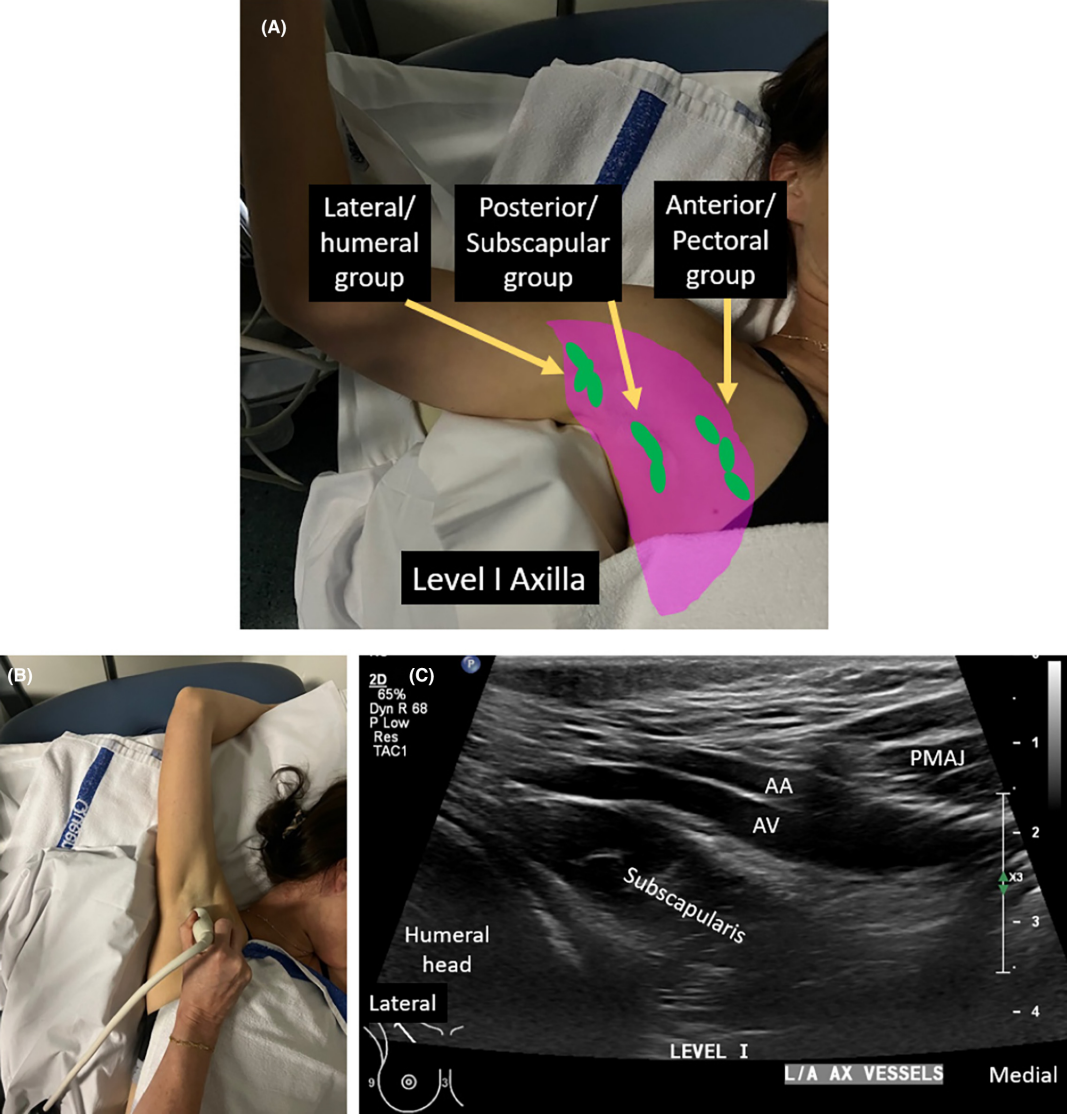
Level I of the axilla. (A). Level I of the axilla outlined by pink. Three groups of lymph nodes are contained within this level which include the anterior/pectoral group, posterior/subscapular group, and the lateral/humeral group. (B). Position of arm and transducer for long‐axis imaging. (C). Sonographic image of Level I the axilla oriented along the long axis of the axillary vessels. AA, axillary artery; AV, axillary vein; PMAJ, pectoralis major muscle.

Level I of the axilla (infero‐lateral or low axilla) incorporates LNs lateral to the lateral border of the pectoralis minor muscle.^17^ It includes LNs from the anterior (pectoral), posterior (subscapular), and lateral (humeral or brachial) groups.^17^ Level I can also include intramammary nodes.^2^ As the largest axillary level, and level in which the majority of sentinel LNs are located, it requires more time to sonographically assess than other levels (Fig. 5).

To image axillary level I, the patient's arm of interest is abducted with their hand above their head. They are often rolled obliquely 45° supported with a foam wedge behind their shoulder and upper chest, to allow easy transducer access to the axilla and lateral chest wall and flattening out of the lateral aspect of the breast and axillary tail. The position of axillary LNs, relative to other structures, with the arm in this position must be appreciated. The anterior/pectoral LN group lie along the infero‐lateral border of the pectoralis minor muscle, along the course of lateral thoracic vessels.^24^ They drain lymph from the upper half of the anterior side of the trunk and major part of the mammary gland.^9^ The axillary tail of spence is usually in contact with these LNs.^17^

The posterior/subscapular group of axillary LNs lie along the posterior axillary fold and inferior border of the subscapularis muscle.^24^, ^25^ They sit medial to the latissimus dorsi muscle prior to it inserting onto the floor of the intertubercular groove of the humerus.^14^ They drain lymph from the upper part of the posterior aspect of the trunk and the axillary tail of the breast.^24^ The lateral/humeral LN group lie along the distal axillary vein, and more towards the humeral shaft, distal to where the latissimus dorsi and teres major muscles attach to the intertubercular groove of the humerus.^26^ The lateral LNs drain lymph from the upper limb, but may also receive drainage from the breast, especially in cases of heavy nodal involvement.^27^

Imaging can begin with the transducer aligned to the long axis of the axillary vessels; referred to as long‐axis imaging. Long‐axis axillary imaging is performed from the anterior to posterior aspects of the axilla, lateral to the lateral border of the pectoralis minor muscle. Imaging is performed at multiple levels including 1. the lateral chest wall level, starting from the inferior aspect of the breast, 2. arm crease level, and 3. humeral shaft level. Relative sonographic and surface body landmarks are used to localise LNs within this level. Anterior superior sonographic landmarks include the pectoralis major muscle. Landmarks at the mid‐level include the axillary vessels, subscapularis muscle, deeper humeral head and shaft, and the glenoid of the scapula. Posterior level I landmarks include the latissimus dorsi and teres major muscles (Video 1).

**Video 1:** Sonographic long and short‐axis real‐time imaging of level I of the axilla (axes are described relative to the axis of the axillary vessels). The video demonstrates transducer positioning and transducer excursion for short‐axis assessment.

**Figure 6:**
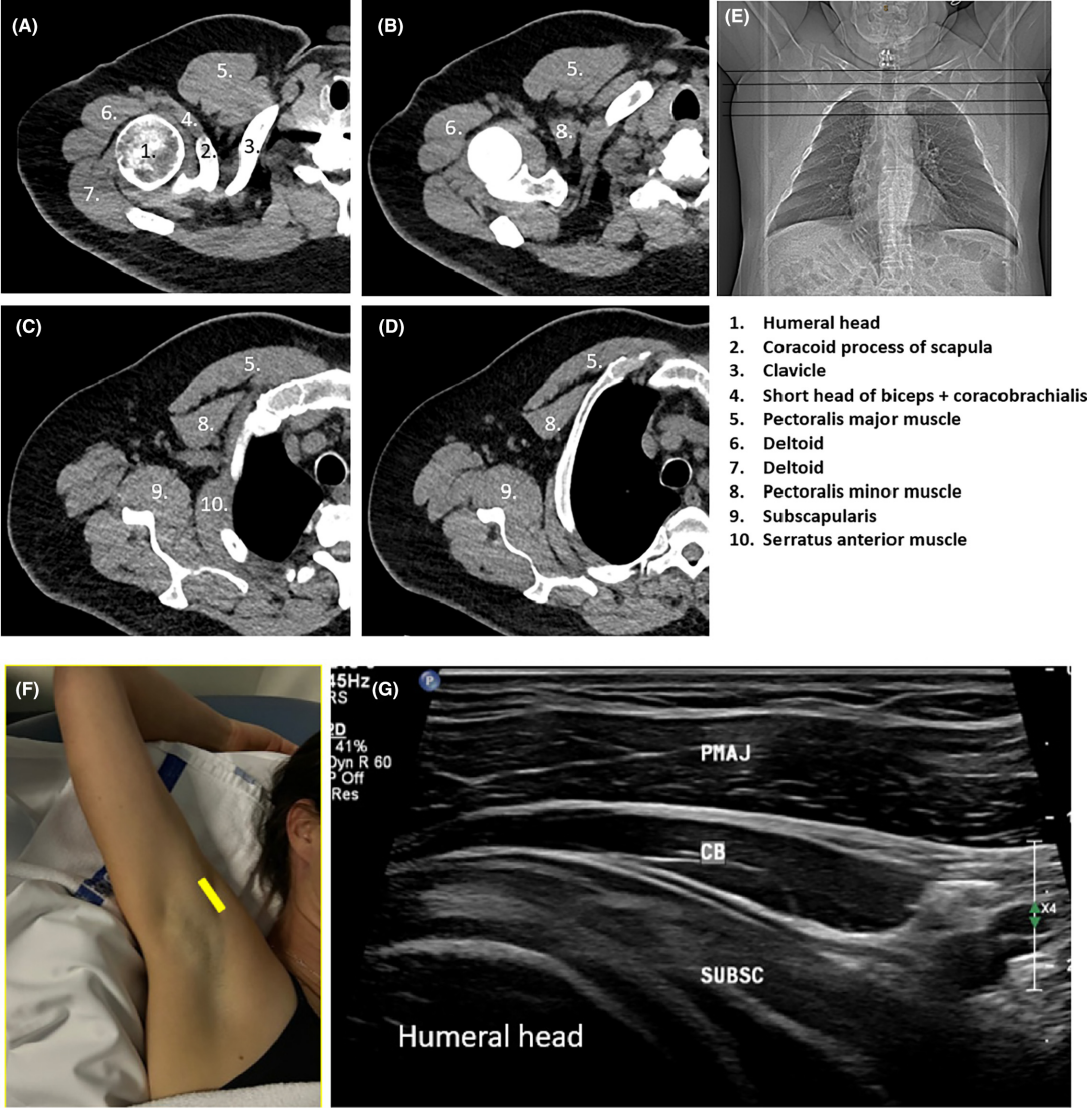
Imaging of the axilla. (A–D) Axial Computed Tomography (CT) images with the patient's arms positioned above their head (as shown in E). (F) Transducer position (yellow line) with arm above head. (G) Long axis sonographic image of the axilla obtained along the anterior axillary fold which demonstrates the coracobrachialis and short head of biceps muscle and tendon in long axis (should not be confused for the pectoralis minor muscle) overlying the humeral head. CB, coracobrachialis muscle; PMAJ, pectoralis major muscle; SUBSC, subscapularis muscle.

It is important anteriorly the coracobrachialis and short head of biceps muscles are not mistaken with the pectoralis minor muscle when the transducer is moved distally towards the arm. The short head of biceps brachii and coracobrachialis muscles sit deep to the pectoralis major muscle when the arm is abducted or positioned above the head. They run into the upper arm, whereas the pectoralis minor muscle is directed to insert onto the ribs (Fig. 6).

**Figure 7:**
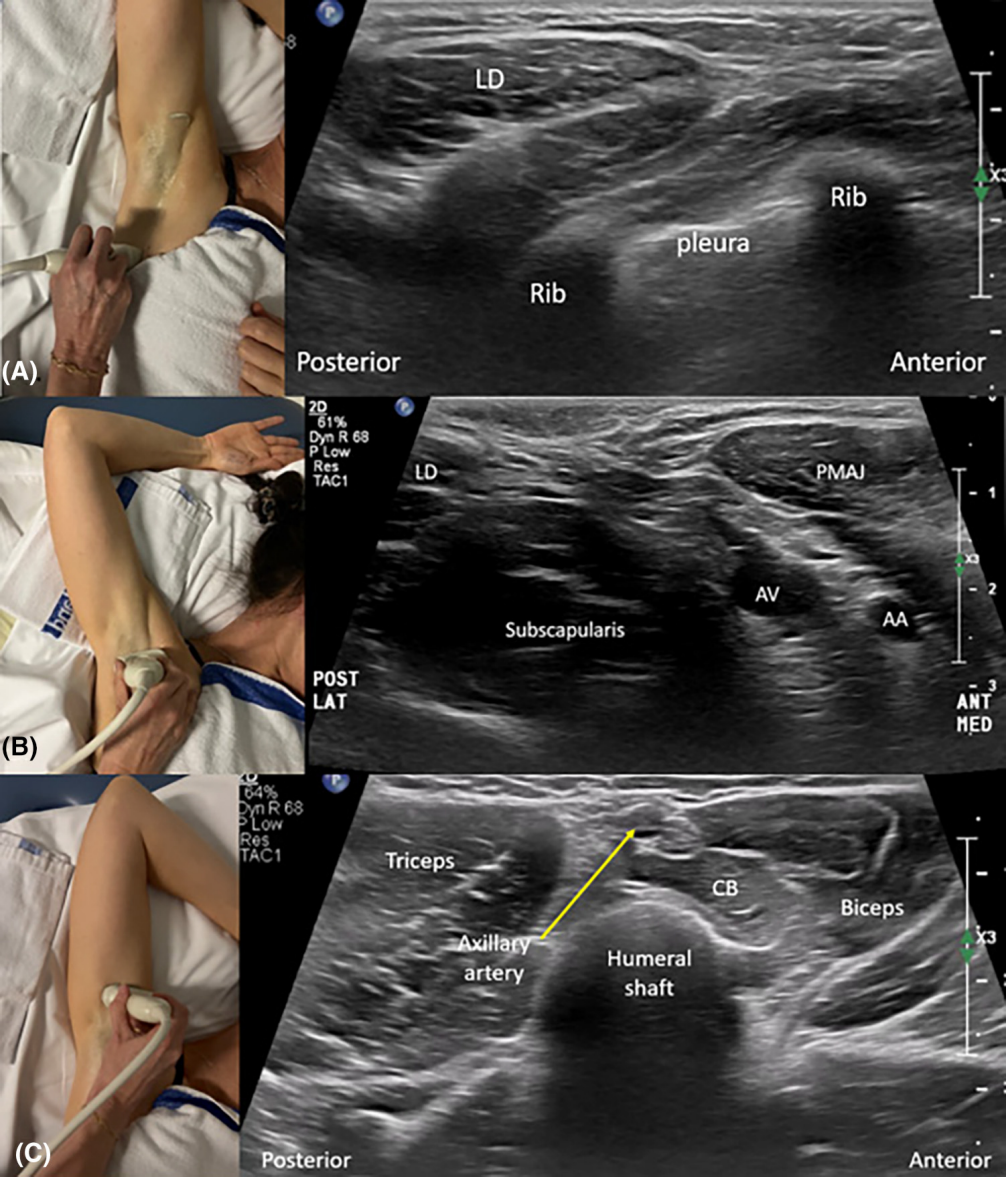
Short‐axis static sonographic image of Level I of the axilla. AA, axillary artery; AV, axillary vein; LD, latissimus dorsi; PMAJ, pectoralis major.

Short‐axis sonographic imaging of surgical level I (relative to axillary vessels) is conducted 1. from the lateral inferior breast, 2. moving superiorly along the lateral chest wall, to 3. the proximal humerus, distal to the latissimus dorsi insertion. The latissimus dorsi muscle is a good sonographic landmark at the posterolateral aspect of the image when starting imaging at the chest wall/inferior breast level. The pectoralis major muscle can be identified at the arm crease level anteriorly. The axillary vessels are identified in the region of the arm crease, between the latissimus dorsi and pectoralis major muscles (Fig. 7).

Cine clips of real‐time sonographic imaging can be performed in short and long axis to document normality or demonstrate the relative positions of multiple suspicious appearing LNs.

### Surgical axillary Level II (mid axilla)

Surgical axillary level II defines axillary LNs between the medial and lateral borders of the pectoralis minor muscle and contains the central group of LNs.^25^ The central group of LNs receive lymph from the anterior, posterior and lateral groups, sit around the pectoralis minor muscle and drain lymph into the apical group of LNs (in level III). The intercostobrachial nerve (ICBN) runs between the LNs within this group. LN enlargement of this group can cause compression of the ICBN resulting in pain along the inner border of the arm.

The space between the pectoralis major and minor muscle fasciae is known as the Rotter space. LNs that sit within the interpectoral space are known as Rotter (interpectoral) LNs.^2^ As Rotter LNs sit deep to the pectoralis major muscle, they are not easily palpated.^28^ Not commonly encountered, breast cancer metastasis rate to Rotter LNs is variably reported, ranging from 4–9.9%.^29^, ^30^ Classified as axillary level II LNs according to the American Journal of Cancer Committee (AJCC) staging system, Rotter LNs can vary in number from one to four.^16^ There is ongoing debate as to whether Rotter LNs should be removed during axillary clearance procedures.^28^

When sonographically imaging surgical axillary level II, the sonographic imaging depth should be set appropriately to allow the interpectoral space to be evaluated. When suspicious Rotter (interpectoral) LNs are identified sonographically, colour Doppler should be utilised to distinguish them from fat and interpectoral vessels (pectoral branches of the thoraco‐acromial artery and venous tributaries) which can also sit in the interpectoral space.^9^ It is important to specifically examine Rotter LNs sonographically, and report suspicious Rotter LNs, as they may not be removed in a typical axillary dissection. In addition, the medial and lateral pectoral nerves can be located in the interpectoral space^24^ (Video 2).

**Video 2:** Long‐ and short‐axis real‐time sonographic imaging of level II of the axilla (axis of scanning is described relative to the axis of the axillary vessels).

**Figure 8:**
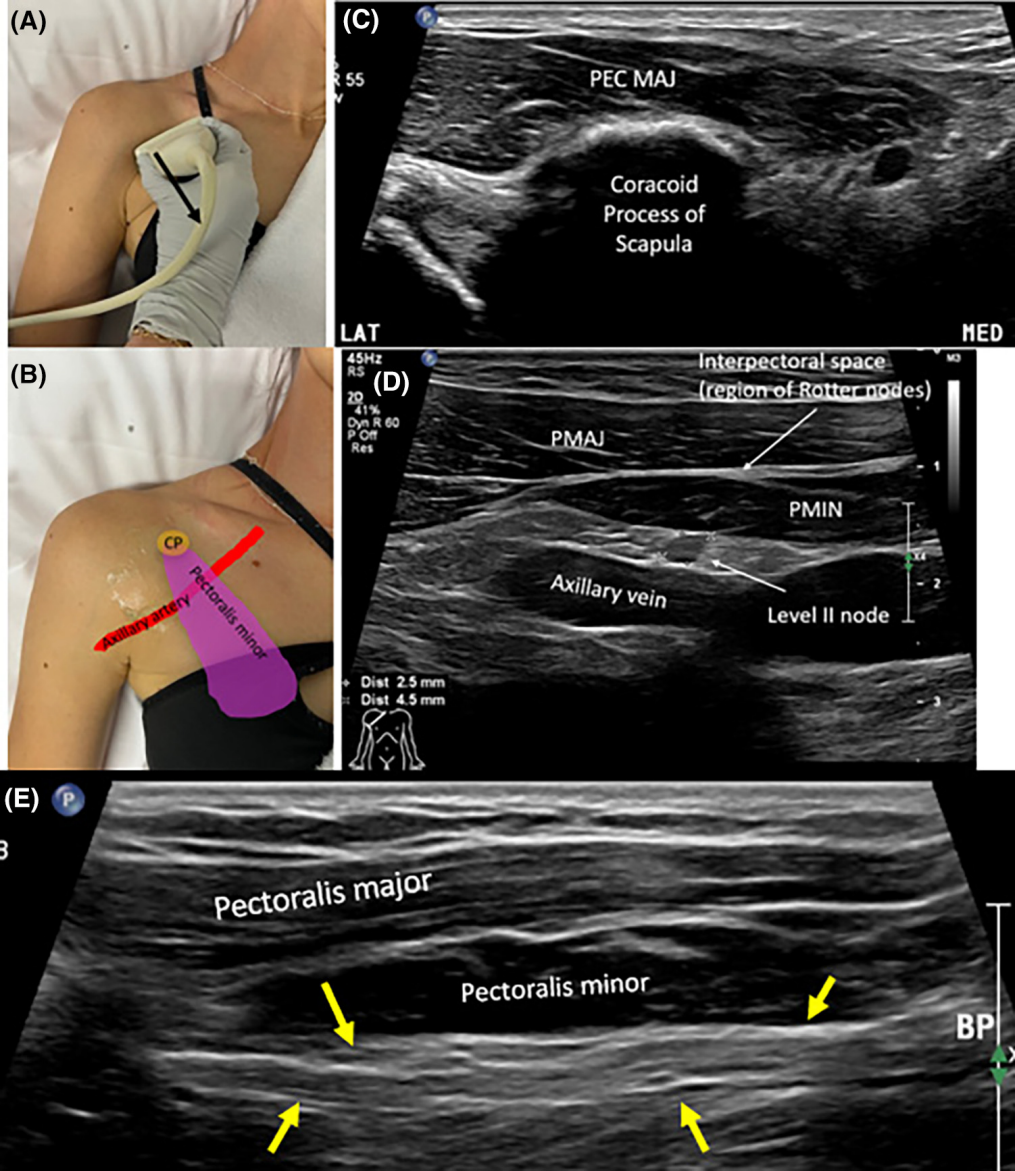
Long‐axis sonographic imaging of level II Axilla (relative to axillary vessels). (A). Transducer position starting over the coracoid process (CP) and scanning inferiorly. (B) Surface anatomy of the region of the coracoid process, pectoralis minor muscle and axillary artery with the arm by the side. (C) Sonographic image of the coracoid process of the scapula. (D) Sonographic image through the pectoralis major and minor muscles, outlining Rotter space, and demonstrating a benign Level II lymph node deep to pectoralis minor muscle. (E) Long‐axis sonographic image of level II outlining the distal portion of the brachial plexus cords (outlined by yelllow arrows) which gives rise to BP branches. BP, brachial plexus; LAT, lateral aspect of image; MED, medial aspect of image; PMAJ and PEC MAJ, pectoralis major muscle; PMIN, pectoralis minor muscle.

Level II can be sonographically assessed with the arm either in the same position as for Level I (arm abducted), or the arm by the side. When the arm is placed by the side, transducer access and sonographic visualisation of level II LNs is improved. Long‐axis imaging is obtained with the transducer oriented along the long axis of the axillary vessels. As the pectoralis minor muscle attaches to the coracoid process of the scapula, long‐axis imaging needs to begin over the coracoid process, and then progress inferiorly through the pectoralis minor muscle to where it attaches to ribs (Fig. 8).

**Figure 9:**
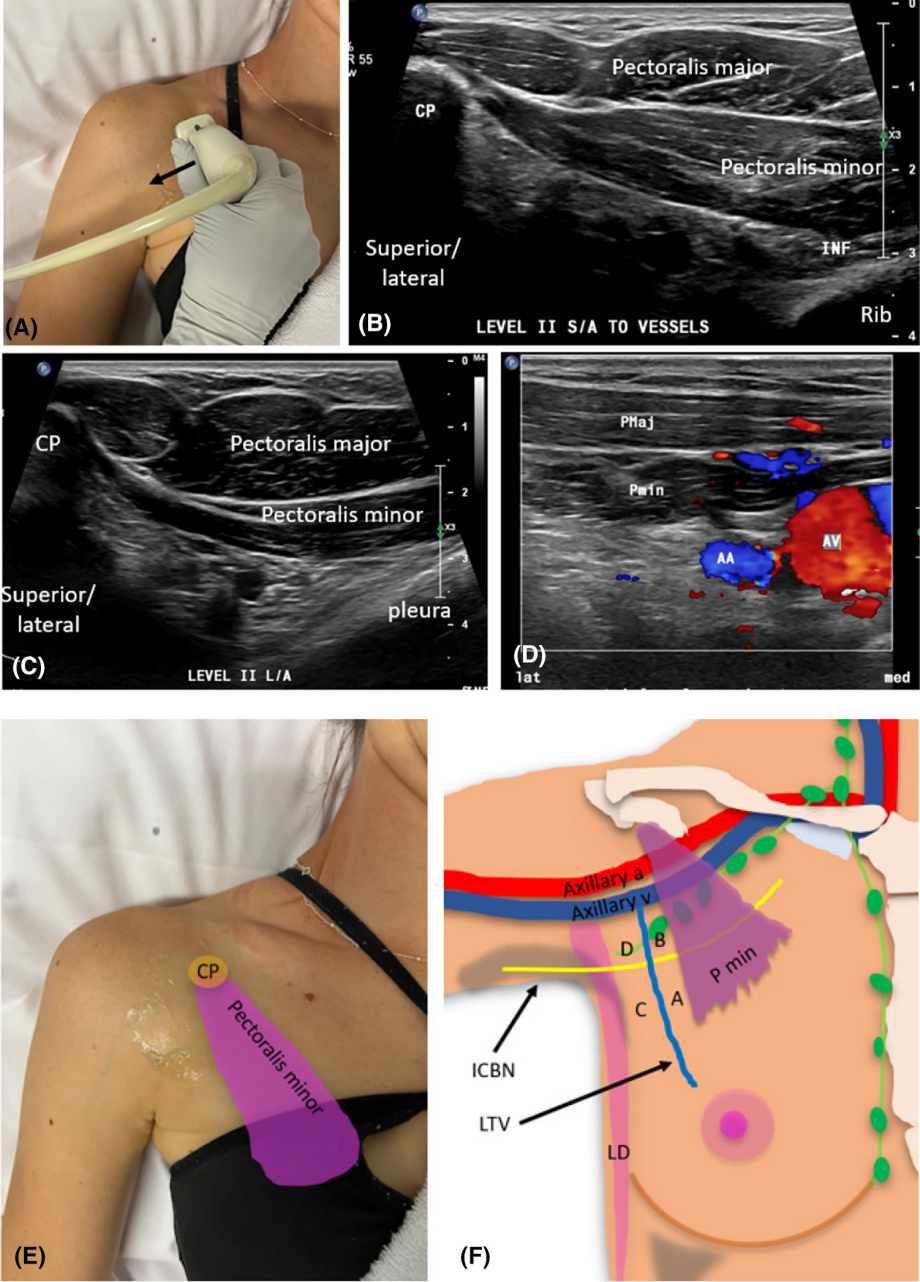
Short‐axis sonographic imaging of axilla level II, relative to the axillary vessels. (A). Transducer and patient positioning (arm by side) demonstrating transducer movement from medial to lateral aspects of level II. The transducer will pass through the region of the pectoralis minor muscle outlined in E, inferior to the coracoid process (CP). (B). Short‐axis image of axillary level II demonstrating the coracoid process superiorly which demonstrates posterior acoustic shadowing. (C). Short‐axis images of axillary level II should demonstrate sufficient depth to allow the axillary vessels and brachial plexus and the deeper pleural cavity to be identified. (D) Short‐axis image which allows axillary and interpectoral vessels including pectoral branches and tributaries of the thoraco‐acromial vessels to be demonstrated with colour Doppler. (E) Surface anatomy of the coracoid process and pectoralis minor muscle. (F) The intersection of the second intercostobrachial nerve (ICBN) and the lateral thoracic vein (LTV) creates four zones of axillary levels I and II (A, B, C, D). INF, inferior aspect of image; lat, lateral aspect of image; LD, latissimus dorsi muscle; med, medial aspect of image; P MAJ, pectoralis major muscle; P min, pectoralis minor muscle; P MIN, pectoralis minor muscle.

For short‐axis sonographic imaging of Level II (relative to the axillary vessels), the superior aspect of the transducer can be placed over the coracoid process, and the pectoralis minor proximal attachment. The transducer is then fanned from the medial aspect of the pectoralis minor muscle through to its lateral aspect (Fig. 9).

### Zones (quadrants) of surgical axillary levels I and Level II

Axillary levels I and the lower part of level II can be subdivided into four quadrants (A, B, C and D). These four quadrants/zones, defined by Clough (2010) further define the location of axillary sentinel LNs.^31^ Zones are divided by the intersection of the horizontally oriented second intercostobrachial nerve (ICBN) and the vertically oriented lateral thoracic vein (LTV), both located lateral to the pectoralis minor muscle.^31^ Zone A is the lower medial quadrant, B: upper medial quadrant, C: lower lateral quadrant, D: upper lateral quadrant.^31^ Up to 98% of SLNs are reported to be located in the medial zones (A and B), adjacent to the LTV.^31^ A much lower percentage of sentinel LNs have been identified in lateral zones. 2–10% sentinel LNs have been identified in zone C and less in Zone D (upper lateral quadrant).^31^

The intercostobrachial nerve (ICBN) is a purely sensory nerve originating from the lateral cutaneous branch of the second intercostal nerve.^32^ It supplies the skin of the 2^nd^ intercostal space at the medial axillary wall, axillary skin and medial upper arm.^33^ It courses through the external intercostal and serratus anterior muscles of the lateral chest (mid axillary line)^32^, obliquely through the axilla to the posteromedial arm where it joins branches of the medial brachial cutaneous nerve.^33^ During its course it can be at risk of iatrogenic injury during procedures including LN dissection, sentinel LN biopsy and mastectomy which can cause sensation changes (pain or paraesthesia) to areas it innervates.^33^ The LTV, a tributary of the axillary vein, drains the breast laterally, then courses vertically towards the axilla before joining the axillary vein anterior to the thoracodorsal vein.^31^

### Surgical axillary level III (apical or infraclavicular LNs)

Level III axillary malignant LNs carry a worse prognosis.^16^ The axillary surgical level III is located medial and superior to the pectoralis minor muscle, inferior to the clavicle and contains infraclavicular (apical) LNs, named as they lie in the region of the axillary apex.^2^ Lying deep to the clavipectoral fascia, they drain lymph from the upper part of the breast and central LNs. Lymph then drains from axillary level III into the subclavian lymph trunk on the right and the thoracic duct on the left.

**Figure 10:**
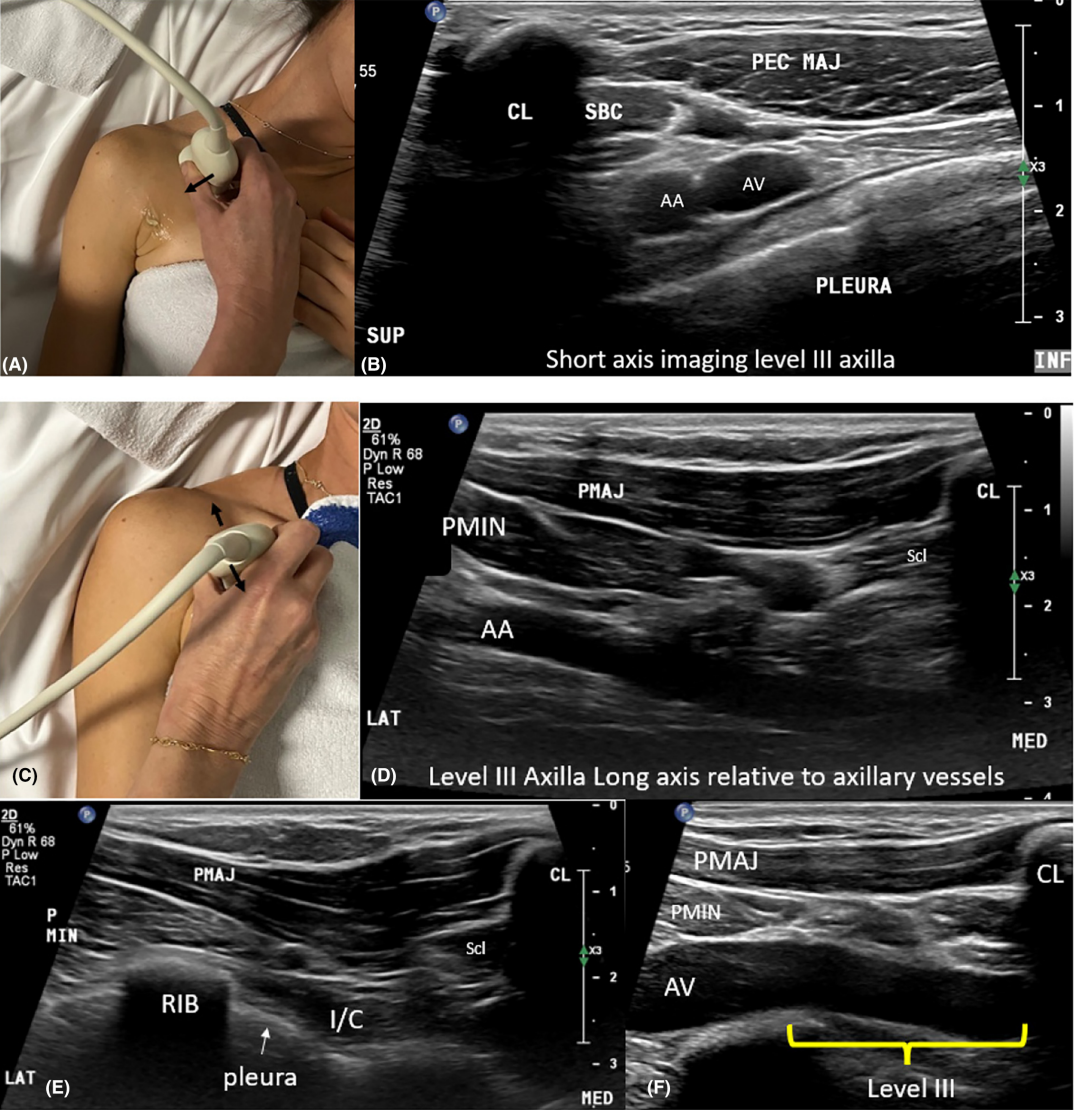
Sonographic imaging of level III of the axilla (Right axilla). (A). Transducer positioning and arm positioning for short‐axis imaging level III. (B). Short‐axis sonographic image of level III of the axilla (axillary vessels seen in cross section). (C). Transducer positioning for long‐axis imaging level III. (D). Long‐axis sonographic image of level III demonstrating the long axis of part 1 of the axillary artery which is located within level III of the axilla. (E). Long‐axis image level III inferior level to the axillary vessels, demonstrating rib, pleura and intercostal (I/C) muscles. (F). Long‐axis image of axillary level III demonstrating the long axis of the axillary vein and outlining the extent of level III of the axilla. AA, axillary artery; AV, axillary vein; CL, clavicle; INF, inferior aspect of the image; LAT, lateral aspect of image; MED, medial aspect of the image; PEC MAJ/PMAJ, pectoralis major muscle; PMIN, pectoralis minor muscle; SBC/Scl = subclavius muscle; SUP, superior aspect of the image.

Sonographic imaging of surgical axillary level III is obtained with the arm by the side, as placing the arm above the head limits transducer access and visibility of this level due to shadowing from the clavicle. Short‐axis imaging (relative to axillary vessels) is obtained by placing the transducer between the clavicle and upper chest, perpendicular to the axillary vessels. The clavicle, pectoralis major muscle, axillary vessels and pleura are used as sonographic landmarks. The transducer is swept laterally until the pectoralis minor muscle comes into view which signposts the start of axillary level II (Fig. 10).

Long‐axis sonographic imaging of level III is obtained by placing the transducer inferior to the clavicle, along the long axis of the axillary vessels. The transducer is fanned superiorly and inferiorly ensuring the space between the medial border of the pectoralis minor muscle and the clavicle is assessed. The transducer is also fanned inferiorly to image ribs and pleura (Video 3).

**Video 3:** Real‐time sonographic short and long‐axis imaging of level III of the axilla.

## Localisation of Axillary Lymph Nodes Using MRI

**Figure 11:**
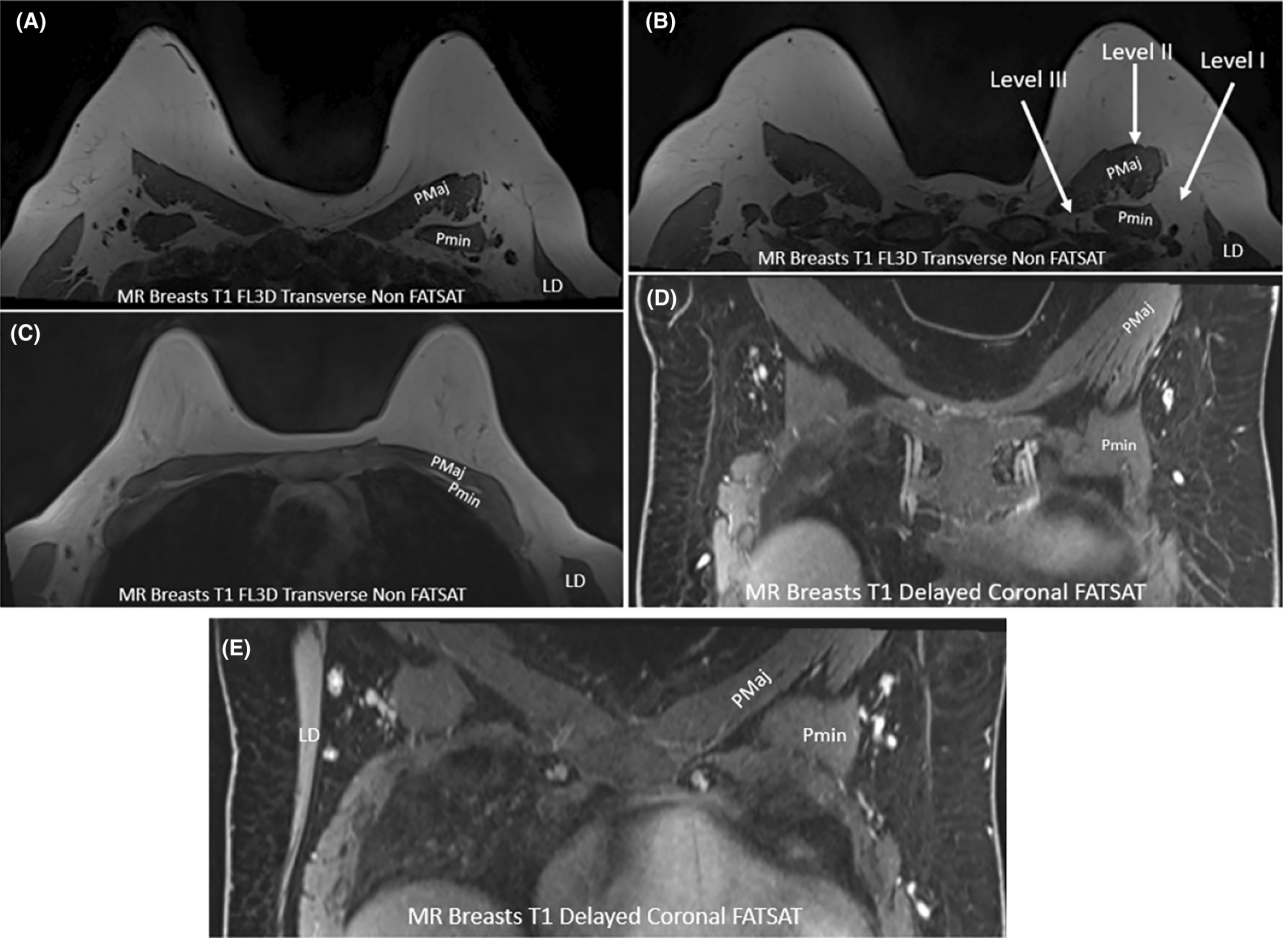
Magnetic resonance (MR) imaging of the breast and axilla. (A–C) Transverse MR images of different breasts, demonstrating variation in the appearances of the pectoral and latissimus dorsi muscles, which can be used as landmarks to identify the three axillary levels. (D, E) Coronal MR images of the chest wall and axilla region to allow identification of the levels of the axilla. (D) is acquired more anteriorly, and (E) is acquired slightly more posteriorly as the latissimus dorsi muscle observed laterally. LD, latissimus dorsi muscle; PMaj, pectoralis major muscle; Pmin, pectoralis minor muscle.

A standard breast MRI can be added to the diagnostic workup of breast cancer patients to further image and delineate breast tumour size and extent, allowing contralateral comparison and imaging in three planes.^34^ Although ultrasound is the primary imaging tool for the axillary region, breast MRI can allow for assessment of LN metastasis.^6^ Breast MR is most useful for demonstrating internal mammary, and supraclavicular LN involvement.^2^ Standard breast MR, using the breast coil, is not as effective at imaging axillary LNs as using a dedicated axilla coil.^6^ The imaging spatial resolution obtained from MR is lower than that of ultrasound which can magnify imaging to a greater degree. MRI and has advantages over ultrasound, including potentially improved visualisation in larger patients, high sensitivity (95% compared to 87% for ultrasound), excellent soft tissue contrast, and less operator dependence.^2^, ^34^ The increased sensitivity of MRI is due to the detection of signal changes in abnormal LNs with diffusion weighted and dynamic contrast enhanced imaging.^35^ When using the breast coil for MR imaging, patients are positioned prone, resting on the breast coil, with both arms above their head. Hence, the arm position is similar to that of sonographic imaging of the breast. Muscular landmarks are similarly used to define the three surgical axillary levels and their LNs (Fig. 11).

The characteristics of suspicious LNs on MR imaging include irregular margins, inhomogeneous cortex, perifocal oedema, asymmetry and absence of fatty medulla (hilum) or chemical shift artefact.^6^

## Breast Cancer Metastasis to Non‐axillary Lymph Nodes

Although the axillary LNs are the main site of breast cancer LN metastasis, further suspicious LNs can be encountered in the supraclavicular, internal mammary and chest wall regions.^36^ Post‐surgically, the direction of lymph flow from the breast to LN basins across the chest wall may be altered. Lymph draining from the breast can occasionally skip axillary LNs, or even axillary and parasternal LNs and drain directly into supraclavicular or parasternal LNs on the contralateral side of the sternum. When metastases skip or jump LN groups, they are called ‘skip’ or ‘jump’ metastases.^36^

Although rare, breast cancer skip metastasis can jump from axillary to supraclavicular LNs.^36^ Supraclavicular LNs are the second most common site of local recurrence of breast cancer after axillary LNs, but recurrence here does not have as good prognosis as axillary LN involvement.^36^ Early detection of supraclavicular LN metastasis however can guide local and systemic treatment and improve overall survival.^37^

Metastatic breast cancer that involves the internal mammary LNs is of similar prognostic importance as axillary LN involvement.^38^ The internal mammary (parasternal) LN chain extends from the first through to the 6^th^ intercostal space.^16^ LNs are largest in the first three intercostal spaces and usually measure less than 6 mm when normal.^20^ The internal mammary LNs provide an lymph drainage pathway from anterior phrenic LNs at the diaphragm into the thoracic venous system on the right and the thoracic duct on the left. It follows the course of the internal mammary artery and vein near the lateral sternal margins. As the internal mammary vein drains into the subclavian vein, lymph also flows from the internal mammary lymph chain to the supraclavicular LNs.^36^ Internal mammary LNs sit relatively deep within the chest wall, and are more difficult to palpate during a clinical examination.^39^ If suspicious LNs are identified sonographically in this location, they are difficult to biopsy, as they can be deep to costal cartilage or the sternum.^39^

## Sonographic Classification of Lymph Node Status

LNs are classified as either appearing benign or suspicious of harbouring metastatic spread. Benign LNs can include normal and reactive LNs. LNs suspicious of malignancy (typically metastatic) are identified sonographically by their qualitative and quantitative (measurement) criteria.

### Benign axillary lymph node sonographic appearance

**Figure 12:**
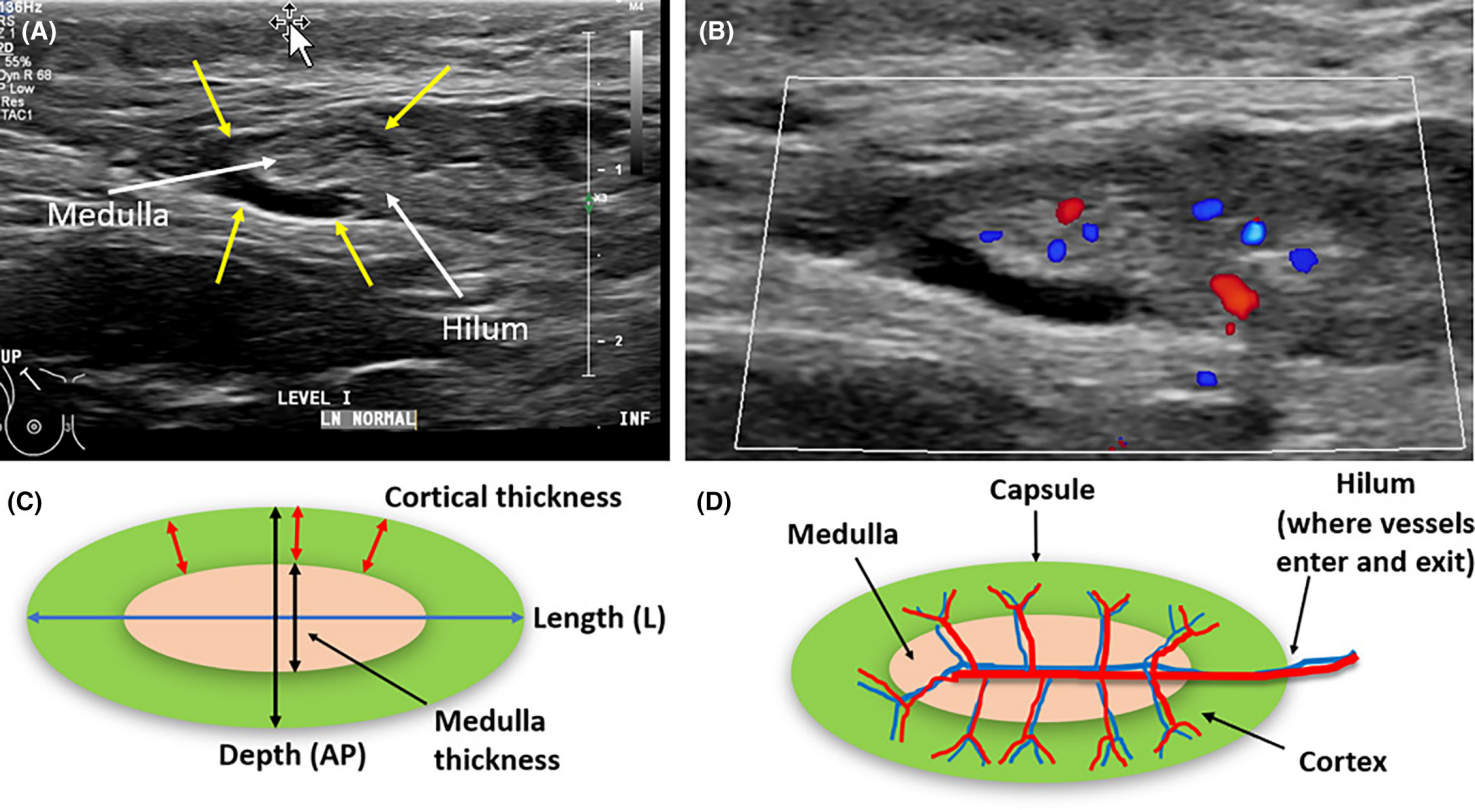
Benign axillary lymph node (LN) appearance. (A) Sonographic long‐axis image of an axillary LN (outlined by yellow arrows). The echogenic medulla and hilum are demonstrated. (B) Sonographic image with colour Doppler demonstrating vessels entering the LN and radiating out from central medulla to the cortex. (C) Diagram of the long axis of the LN outlining the anteroposterior (AP) depth and measures. (D) Components of the LN outlined.

Benign LNs appear oval or reniform (kidney) shaped.^40^ Typically short‐axis measures of LNs are used to classify LNs as benign or suspicious, however, more specific criteria are used for axillary LNs.^25^ The length of a benign LN, determined from a long‐axis image should typically be at least two times greater than the depth.^41^ The LN cortex should appear homogenous in thickness and echogenicity throughout^41^ (Fig. 12).

For axillary LNs, cortical thickness up to 3 mm is considered benign.^42^, ^43^ The cortex should be hypoechoic relative to an echogenic medulla and can be isoechoic to surrounding tissues.^41^ The cortical margin should be smooth, regular and the cortex easily distinguished from the medulla.^44^, ^45^ If an echogenic medulla predominates, it can be difficult to distinguish a LN from surrounding axillary fat, and it is almost always benign.^16^ The LN hilum may or may not demonstrate increased echogenicity. Vessels entering the LN hilum may be identified branching out radially or perpendicular to central flow (radial distribution) with colour or power Doppler.^43^ Reactive benign LNs are generally oval, but can demonstrate some cortical thickening, thinning, and increased echogenicity, but should maintain their echogenic medulla.

### Suspicious axillary lymph node sonographic appearance

**Figure 13:**
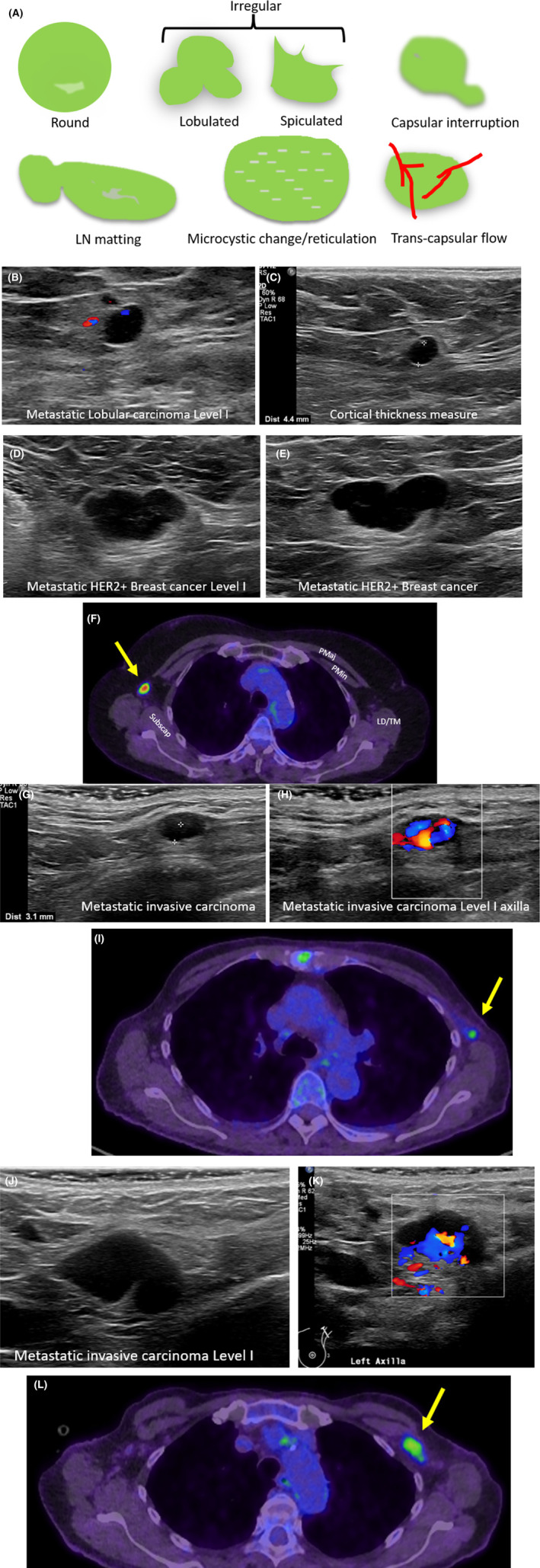
Metastatic axillary lymph nodes. (A) Descriptions used for defining suspicious axillary lymph nodes (LNs). (B) Sonographic images of axillary level I metastatic LN, confirmed with biopsy and histological analysis. The LN is rounded in shape and hypoechoic. (C) This axillary metastatic LN has asymmetric cortical thickness and demonstrates a loss of the echogenic medulla. Cortical thickness is 4.4 mm. (D, E) Biopsy confirmed metastatic LN in axillary level I in patient HER2 positive breast cancer. (F) Metastatic axillary level I LN demonstrated on sonographic and axial PET CT images (yellow arrow) in patient with HER2 positive breast cancer. The LN is lobulated in shape and demonstrates asymmetric cortical thickening. On the axial PET CT image, the node can be identified lateral to the pectoralis minor muscle, consistent with level I of the axilla. (G–L) Biopsy confirmed metastatic axillary LNs (Level I), demonstrated in two different patients. LNs sonographically demonstrate transcapsular flow in both patients. LD, Latissimus dorsi muscle; PET CT, positron emission tomography computed tomography; PMaj, pectoralis major muscle; PMin, pectoralis minor muscle; Subscap, subscapularis muscle; TM, teres major muscle.

Suspicious LNs sonographically appear round or irregular (lobulated or spiculated) in shape.^2^, ^46^ A long‐to‐short axis ratio that approaches 1 is suspicious of LN metastases.^47^ Axillary LNs with a cortical thickness ≥3 mm are suspicious of malignancy.^48^ This cortical measurement is specific for axillary LNs.^43^ LN cortical thickening can be defined as homogenous, eccentric or throughout the whole cortex.^49^ Asymmetric cortical thickening or bulging is suspicious^2^, ^50^ (Fig. 13).

The capsule of suspicious LNs on sonographic imaging may appear blurred or interrupted, either focally or diffusely, and can be matted.^49^ Partial matting results in LNs in contact, and complete matting results in LNs unable to be individually distinguished from each other. Diffuse or focal LN echogenicity changes such as hyperechoic deposits or cystic areas within the cortex or medulla are suspicious findings.^50^ Diffuse cortical microcystic changes, termed reticulation, may sonographically appear as multiple hyperechoic lines or echogenic areas and indicate a suspicious LN.^43^ When the LN medulla and hilum, collectively are not visualised sonographically (are completely hypoechoic) the LN is termed suspicious.^50^ Evidence of transcapsular flow using colour or power Doppler, where vessels enter or perforate the LN in areas other than the hilum is considered suspicious.^43^ In addition, more than one suspicious appearing axillary LN raises the suspicion of metastatic breast cancer spread.^49^

## Conclusion

Assessment of axillary LN metastatic involvement plays a crucial role in determining treatment strategies and predicting outcomes in breast cancer patients. Sonographic imaging is used to identify and accurately report the presence, number, and location of suspicious axillary or regional breast LNs. An understanding of the axillary anatomy, surgical axillary levels, sonographic landmarks, and the sonographic technique to assess and localise axillary LNs is required. Additionally, the sonographic criteria to discriminate between benign and suspicious axillary LNs, which is slightly different to other LNs is essential to appreciate to allow consistent reporting of axillary LN status. Accurate diagnosis and localisation of suspicious axillary LNs can allow for appropriate patient management and treatment, including any surgical planning.

## Conflict of Interest

The authors declare no conflict of interest.

## Data Availability

Data sharing not applicable to this article as no datasets were generated or analysed during the current study.
